# Gut Microbiota Profiles and Microbial-Based Therapies in Post-operative Crohn's Disease: A Systematic Review

**DOI:** 10.3389/fmed.2020.615858

**Published:** 2021-01-28

**Authors:** Xiaojun Zhuang, Zhenyi Tian, Na Li, Ren Mao, Xiaozhi Li, Min Zhao, Shanshan Xiong, Zhirong Zeng, Rui Feng, Minhu Chen

**Affiliations:** Department of Gastroenterology, The First Affiliated Hospital, Sun Yat-sen University, Guangzhou, China

**Keywords:** crohn's disease, mucosa-associated microbiota, feces-associated microbiota, post-operative recurrence, microbial-based therapies

## Abstract

**Background and Aims:** Gut microbiota recolonization after intestinal resection had been reported to be associated with post-operative recurrence in Crohn's disease (CD). However, the results of different studies are inconsistent and even contradictory. In addition, knowledge on the efficacy of microbial-based therapies in preventing post-operative recurrence of CD is limited. Therefore, the aim of this review was to investigate gut microbiota profiles in patients with CD before and after surgery and evaluate microbial-based therapies in preventing post-operative recurrence.

**Methods:** Electronic databases were searched from inception to 31 June 2020 using predefined terms. Studies that investigated gut microbiota pre- and post-intestinal resection, and microbial-based therapies in preventing post-operative recurrence, were eligible. Study quality was assessed using either the Newcastle–Ottawa scale or Jadad scoring system.

**Results:** Twelve studies investigating gut microbiota of CD patients suffering from operation, and other 12 studies evaluating the efficacy of antibiotics and probiotics, were included in our review. The mucosa-associated microbiota in surgical biopsy of CD patients is significantly distinct from that in normal mucosa from healthy subjects. Gut microbiota recolonization following surgery might be associated with post-operative recurrence in CD patients. Furthermore, CD patients with post-operative recurrence presented a gain in pro-inflammatory pathogenic bacteria and a loss in short-chain fatty acid-producing bacteria before and after surgery. However, no consistent bacteria or metabolites were found to predict the post-operative recurrence of CD. Additionally, microbial-based therapies are deficient and present restricted widespread clinical utility due to several deficiencies.

**Conclusion:** Recurrence-associated bacteria observed pre- and post- operation might be promising in preventing the post-operative recurrence of CD. Furthermore, potential microbe biomarkers for predicting subsequent disease recurrence should be validated with larger sample sizes using more rigorous and standardized methodologies.

## Introduction

Crohn's disease (CD) is a chronic relapsing inflammatory bowel disease (IBD) with multifactorial pathogenesis and is characterized by recurrent transmural inflammation (1). Eventually, the recurrent inflammation can lead to intestinal stricture and fistulae (often perianal) complications or the creation of abscesses (2). Surgical resection is required in ~70–80% of CD patients, owing to the penetrative nature of the disease, the development of structural changes, and the failure of medical therapy (3–5). However, operative management is not curative, and up to 75% of CD patients will experience post-operative disease recurrence (clinical, endoscopic, or surgical recurrence) over time (6–8). As a consequence, ~30% of patients require a second surgical resection within 5 years (9, 10). Given the significant recurrence risk after surgical resection in CD, elucidating the specific factors that predispose patients to post-operative CD recurrence is a high priority. Multiple clinical risk factors, including active smoking, perforating disorders, prior resection, myenteric plexitis, younger age of disease onset, short disease duration, CD behavior, histologic involvement of resection margins, remnant disease post-operation, and length of the resected segment, have been associated with post-operative CD recurrence; however, these factors are far from being adequate in predicting disease recurrence (11–13).

Gut microbiota alterations have been identified as key contributors to the pathogenesis of CD; the crucial link between gut microbiota dysbiosis and post-operative disease recurrence has been documented by numerous studies (14–16). However, the post-operative role of microbial communities in patients with CD remains unknown, largely owing to heterogeneous studies with highly diverse results. In order to facilitate the use of gut microbiota in improving the diagnosis and treatment of post-operative CD, it is imperative to elucidate the bacteria that are associated with disease recurrence or its absence and evaluate whether these microbial factors could predict the post-operative CD recurrence.

Although there is compelling evidence pointing to a critical role of gut microflora in the post-operative CD recurrence, microbial-based therapies for preventing CD recurrence following surgery remain limited (17). Antibiotics and probiotic supplements aimed at altering gut microbiota composition have both been studied in terms of their ability to prevent post-operative disease recurrence; however, there is currently no evidence-based consensus on the topic (18–20). Antibiotics may be the most cost-effective strategy to prevent post-operative disease recurrence in patients who can tolerate the treatment, but the long-term effects beyond antibiotic cessation are questionable (10). In addition, the prolonged administration of these antibiotics is not feasible, owing to a high rate of side effects, significant toxicity, and bacterial resistance (18). Accumulating evidence has implicated that manipulating gut microflora with probiotics is an appealing alternative in reducing the postsurgical CD relapse rate by counterbalancing harmful bacteria (21). However, there are currently design limitations in probiotic trials for the prevention of post-operative CD recurrence; these limitations include small sample sizes, short observation periods, or the co-administration of other drugs.

The aim of this review was to summarize the results of studies investigating gut microbiota alterations in CD individuals suffering from operative management and to evaluate whether specific gut microbiota variations are associated with the post-operative recurrence (PR) of the disease. Furthermore, we revisited previous randomized controlled trials and high-quality uncontrolled studies in an effort to better elucidate the role of microbial-based therapies in preventing the PR of CD.

## Methods

### Search Protocol

The protocol for this systematic review was registered on the International Prospective Register of Systematic Reviews (PROSPERO) with the ID number CRD42020200956, and the Preferred Reporting Items for Systematic Review and Meta-Analyses (PRISMA) checklist was used as a guideline. A comprehensive search was performed on public databases, including PubMed, Web of Science, Embase, Scopus, and the Cochrane Library (last search: 31 June, 2020), with no date or language restrictions. The MeSH term and free-text word combinations that we used were the following: “Crohn's disease,” “CD,” “post-operative,” “surgery,” “resection,” “recurrence,” “microbiota,” “microbiome,” “microflora,” “bacterial flora,” “antibiotic,” “probiotic.” Boolean operators (AND, OR, NOT) were used to widen and narrow the search results.

### Eligibility Criteria and Study Selection

The articles were selected on the basis of certain criteria: observational studies that focused on gut microbiota profiles associated with the post-operative disease course in CD patients or clinical trials that evaluated the effect of microbial-based therapies (antibiotics and probiotics) on the prevention of the PR of CD. The microbial communities in these studies were assessed from either fecal or mucosal samples. Two independent investigators screened titles and abstracts from the databases according to the eligibility criteria. Subsequently, the included articles were subjected to whole-paper reading, and the accompanying references were checked to identify additional potentially eligible articles. Any discrepancies between the investigators were resolved through discussion until consensus was reached, and a third reviewer was involved if necessary.

### Data Extraction and Quality Assessment

For studies investigating gut microbiota profiles in the PR of CD, we extracted demographic, clinical, and bacterial richness and diversity and taxonomic bacterial composition (phylum, class, order, family, and genus), as well as information on the methodology applied to the microbiota analysis. For each of the studies that evaluated the effect of microbial-based therapies on preventing the PR of CD, we extracted data on the first author of the study, the year of publication, the population examined, intervention details, treatment duration, recurrence definition, follow-ups, and the outcomes.

The quality of the included studies was evaluated using the Newcastle–Ottawa Scale (NOS) for cohort studies (22). The NOS contains three criteria: selection (representativeness of the exposed cohort, selection of the non-exposed cohort, ascertainment of exposure, demonstration that the outcome of interest was not present at start of study), comparability (comparability of cohorts on the basis of the design or analysis), and exposure (assessment of outcome, whether follow-up was long enough for outcomes to occur, adequacy of follow-up of cohorts). A quality score ranging from 0 to 9 was obtained by the use of a rating algorithm previously described: 0–3 (poor), 4–6 (moderate), and 7–9 (high).

The Jadad quality scoring system, which is based on randomization, blinding, and dropouts (withdrawals), was used to assess the quality of the randomized controlled trials (23). The quality scale ranges from 0 to 5 points, with a score of ≤2 indicating a low-quality report and a score of ≥3 indicating a high-quality report.

## Results

### Study Selection and Quality Assessment

**Figure 1:**
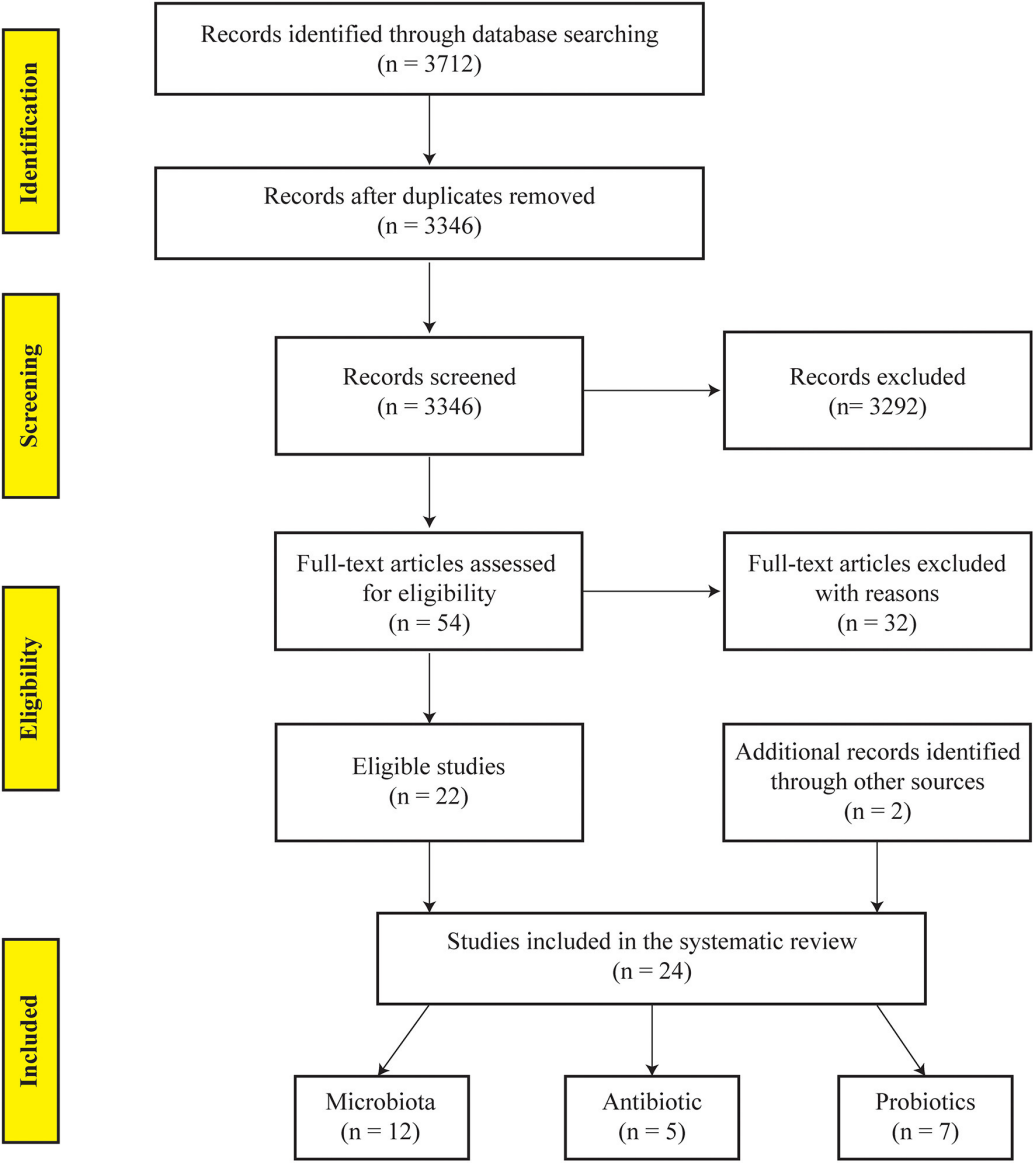
PRISMA flow diagram summarizing the studies identified during the selection process.

The initial database search yielded 3,712 records. After the automatic removal of duplicate, 3,346 unique abstracts were screened and 54 records were selected for full-text review. Finally, 24 original articles were included in this systematic review (Figure 1). These articles included 12 studies reporting gut microbiota profiles in post-operative, and five and seven studies evaluating the efficacy of antibiotics and probiotics, respectively, in preventing the PR of CD (24–47). The quality scores of the included studies were assessed and are reported in Supplementary Table 1.

### Characteristics of Studies Investigating Gut Microbiota in Post-operative CD

The demographic and clinical characteristics of the patients from the included studies investigating gut microbiota are detailed in Table 1. The studies were performed in different geographical regions (including Sweden, Belgium, France, Canada, Australia, France, and the USA) from 2002 to 2020, and almost all of their subjects were adults. In patients with CD, either ileal or ileocecal resections were performed to remove diseased areas from the ileum and right colon with ileocolonic anastomosis. Approximately, 3–18 months after intestinal surgery, a post-operative colonoscopy was performed to assess the endoscopic recurrence based on the Rutgeerts score (48); and the PR was assigned a Rutgeerts score of ≥i2. In addition, the endoscopic recurrence rates were reported as ranging between 33.3 and 73.7% in the included studies.

**Table 1:** Characteristics of studies investigating gut microbiota profiles in patients with CD.

**Studies**	**Country**	**Year**	**Sample size**	**Gender (male %)**	**Age**	**Resection**	**Ileocolonoscopy time**	**Recurrence evaluation**	**Endoscopic recurrence**	**Medicine before resection**	**Medicine after resection**	**Follow-up time**	**RR (%)**
Hamilton et al.	Australia	2020	CD, *n =* 130	44	M: 36	Ileocecal resection	6 and 18 m	Endoscopic recurrence	Rutgeerts score	–	Metronidazole (23), Thiopurine (72), Adalimumab (35)	6, 12, 18 m	6 m: 34.78 18 m: 42.86
Strömbeck et al.	Sweden	2020	CD, *n =* 21	66.7	R: 17–63	Ileocecal resection	52 (41–58) w	Endoscopic recurrence	Rutgeerts score	No medication (4), 5-ASA (7), Corticosteroids (13), Thiopurines (10), Anti-TNF (1)	No medication (8), 5-ASA (8), Corticosteroids (2), Thiopurines (6)	3–10 w; 1 y	1 y: 38.1
			Ctrl, *n =* 7	42.9	R: 20–36								
Machiels et al.	Belgium	2020	CD, *n =* 120	47.5	–	Ileocecal resection	6 m	Endoscopic recurrence	Rutgeerts score	Corticosteroids (25), Anti-TNF (22), Immunosuppressants (24), Antibiotics (21)	Thiopurines (6), Anti-TNF (10), Vedolizumab (1)	1, 3, 6 m	6 m: 43
			Ctrl, *n =* 39	–	–								
Sokol et al.	France	2019	CD, *n =* 201	49	M: 34.6	Ileal resection	6–12 m	Endoscopic recurrence	Rutgeerts score	Steroids (65), Immunosupressant (63), Anti-TNF (95), Antibiotics (68)	Immunosupressant (48), Anti-TNF (68), Antibiotics (13)	6–12 m	6–12 m: 50
Keshteli et al.	Canada	2018	CD, *n =* 38	34.2	R: 18.6–66	Ileocolonic resection	6–12 m	Endoscopic recurrence	Rutgeerts score	–	No medication (12), 5-ASA (7), AZA/6-MP (13), MTX (5), Corticosteroids (2), Adalimumab (4), Infliximab (6)	6–12 m	6–12 m: 73.7
Laffin et al.	Canada	2018	CD, *n =* 45	37.8	M: 43.2	Ileocolonic resection	6 m	Endoscopic recurrence	Rutgeerts score	Steroids (15), Biologic therapy (22), 5-ASA (6), AZA (17), MTX (5)	Steroid (2), Biologic therapy (25), 5-ASA (5), AZA (16), MTX (3), Antibiotics (18)	6 m	6 m: 33.3
Wright et al.	Australia	2017	CD, *n =* 34	41	R: 23–43	Ileal and ileocecal resection	6 and/or 18 m	Endoscopic recurrence	Rutgeerts score	–	Metronidazole (6), Thiopurine (22), Adalimumab (6)	6 and/or 18 m	6 m: 37.0 18 m: 58.3
			Ctrl, *n =* 12	33	R: 46–83								
Mondot et al.	France	2015	CD, *n =* 20	–	–	Ileocolonic resection	6 m	Endoscopic recurrence	Rutgeerts score	Antibiotics (0)	*Lactobacillus johnsonii* LA1	6 m	6 m: 50
Cruz et al.	Australia	2014	CD, *n =* 12	58.3	R: 19–50	Ileocecal resection	6 m	Endoscopic recurrence	Rutgeerts score	Antibiotics (0), Probiotics (0)	No medication (2), Thiopurine (7), Adalimumab (3)	6 m	6 m: 50
			Ctrl, *n =* 10	50	R: 26–66								
Dey et al.	USA	2013	CD, *n =* 6	50	M: 34.6	Ileocolic resection	5–10 m	Endoscopic recurrence	Rutgeerts score	5-ASA (1), Anti-TNF (5), Antibiotics (4), Anti-4-integrin (1), Probiotics (5)	No medication (1), Adalimumab (4), Certolizumab (1)	5–10 m	5–10 m: 50
			Ctrl, *n =* 20	20	M: 55.2								
Sokol et al.	France	2008	CD, *n =* 21	–	–	Ileocolonic resection	6 m	Endoscopic recurrence	Rutgeerts score	–	*Lactobacillus johnsonii* LA1, Placebo	6 m	6 m: 61.9
Neut et al.	France	2002	CD, *n =* 61	36.1	R: 19–68	Ileocolic resection	3 m, 1 y	Endoscopic recurrence	Rutgeerts score	Cefoxitin (13) at the time of surgery	No medication	3 m, 1 year	3 m: 42.9
			Ctrl, *n =* 10	–	M: 62								1 years: 65
Total			CD, *n =* 709 Ctrl, *n =* 98				3–18 m						33.3–73.7

*CD, Crohn's disease; Ctrl, control; M, median; R, range; m, month; y, year; 5-ASA, 5-aminosalicylic acid; AZA, azathioprine; 6-MP, mercaptopurine; MTX, methotrexate; TNF, tumor necrosis factor; RR, recurrence rate*.

*“–”: No information provided*.

### Handling and Analysis of Samples

Mucosal and/or fecal samples were collected at the time of surgery or at different time points in the follow-up period. The various differences in the handling and analysis of samples that were observed among the individual studies are shown in Supplementary Table 2. In the majority of the studies, the samples were preserved at either −20 or −80°C. In approximately half of the studies, DNA extraction was used for microbiota analysis, using reliable kits from different manufacturers, while other studies did not provide information on the methods they used. Most of the analyzed studies employed sequencing techniques based on the hypervariable regions of the 16S ribosomal ribonucleic acid (rRNA) gene for the gut microbiota analysis. However, two early studies analyzed specific bacterial strains using fluorescence *in situ* hybridization (FISH) and culture-based methods. In addition, taxonomic classification was assigned via multiple databases, with Greengenes and Silva being the most commonly used ones.

### Mucosa-Associated Microbiota Profiles in Surgical Biopsy Samples of CD Patients

In order to characterize the mucosa-associated microbiota in CD patients, we compared the surgical biopsies of CD patients and the normal samples from healthy subjects in Table 2. With regard to the microbiota community diversity, the alpha-diversity of mucosa-associated microbiota from surgical specimens decreased significantly in CD patients. In addition, beta-diversity analysis revealed that the mucosa-associated microbiota in the surgical biopsies of CD patients deviated significantly from those of healthy controls.

**Table 2:** Mucosa-associated microbiota of patients with CD from the resection specimen at the time of surgery compared to healthy controls.

**Studies**	**Wright et al**.	**Dey et al**.	**Neut et al**.	**Machiels et al**.	**Cruz et al**.	**Total** ***↑↓***	
**α-Diversity**							
Richness	↓	↓	–	↓	↓	0	4
Diversity	↓	↓	–	↓	↓	0	4
**β-Diversity**	D	D	–	D	D	D = 4	
**PHYLUM**							
Bacteroidetes	↓	↓				0	2
Proteobacteria	↑	↑		↑		3	0
**FAMILY**							
*Bacteriodaceae*	↓	↓				0	2
*Clostridiaceae*	↑			↑		2	0
*Enterobacteriaceae*	↑	↑				2	0
*Enterococcaceae*	↑			↑		2	0
*Lachnospiraceae*	↓			↓		0	2
**GENERA**							
*Actinomyces*	↑				↑	2	0
*Bacteroides*	↓	↓	↓		↓	0	4
*Bifidobacterium*	↑		↓		↓	1	2
*Blautia*				↓	↓	0	2
*Clostridium*			↓		↓	0	2
*Coprococcus*				↓	↓	0	2
*Dorea*				↓	↓	0	2
*Enterococcus*	↑		↑		↑	3	0
*Eubacterium*			↓	↑	↓	1	2
*Faecalibacterium*	↓			↓	↓	0	3
*Fusobacterium*	↑	↑			↑	3	0
*Lachnobacterium*	↓			↓	↓	0	3
*Lachnospira*	↓			↓	↓	0	3
*Lactobacillus*	↑				↑	2	0
*Odoribacter*	↓				↓	0	2
*Paraprevotella*				↓	↓	0	2
*Phascolarctobacterium*	↓			↓		0	2
*Prevotella*			↓	↓		0	2
*Ruminococcus*	↓	↓	↓	↓	↓	0	5
*Streptococcus*	↑				↑	2	0
*Veillonella*	↑				↑	2	0

*Only bacteria, for which at least two studies have reported the alterations at different taxonomic levels (phylum, family, and genera), are displayed in the table*.

*↑ = higher α-diversity or bacteria are more abundant, in CD compared to control; ↓ = lower α-diversity or bacteria are less abundant, in CD compared to control; D = bacterial β-diversity differ between CD patients compared to controls. N = no difference in β-diversity. – = no information provided. Red box represents an increased level, green box represents a decreased level, and empty box represents that no comparison was reported in the included studies*.

A comparison of the relative bacterial abundance at different taxonomic levels revealed that the mucosal microbiome composition of CD patients and control cases differed in a manner of ways. At the phylum level, the relative abundance of Bacteroidetes and Firmicutes in surgical biopsies of CD patients at the time of resection decreased; on the contrary, the relative abundance of Proteobacteria and Fusobacteria increased. At the family level, surgical specimens from CD patients contained an increase in *Clostridiaceae, Enterobacteriaceae*, and *Enterococcaceae* populations and a decrease in *Bacteroidaceae* and *Lachnospiraceae* populations. Moreover, in CD patients who suffered from mucosal microbiota dysbiosis, which also showed mucosal microbiota dysbiosis at the deeper genus level, 21 bacterial genera were found in different abundances. More specifically, the *Actinomyces, Enterococcus, Fusobacterium, Lactobacillus, Streptococcus*, and *Veillonella* populations increased, while the *Bacteroides, Blautia, Clostridium, Coprococcus, Dorea, Faecalibacterium, Lachnobacterium, Lachnospira, Odoribacter, Paraprevotella, Phascolarctobacterium, Prevotella*, and *Ruminococcus* populations decreased. However, the changes in the *Bifidobacterium* and *Eubacterium* numbers reported from surgical biopsies are divergent and even contradictory in the included studies. A more comprehensive list of specific mucosa-associated microbiota alterations of CD patients at the phylum, class, order, family, and genus levels is presented in Supplementary Table 3.

### Dynamic Alterations of Gut Microbiota During the Postsurgical Disease Course

Intestinal resection may play a crucial role in the gut microbiota recolonization process. Several studies have investigated temporal alterations of the mucosal microbiota community structure in CD patients before and after surgery (23–34). The taxonomic differences in the mucosa-associated microbiota of CD patients following surgery compared to baseline are presented in Table 3 and Supplementary Table 4. There were no significant alpha-diversity differences between the surgical samples and those obtained from the post-operative follow-up. In addition, it was observed that the beta-diversity of the gut microbiota of post-operative CD patents differed from that at the time of resection in three studies, whereas one study reported no significant differences.

**Table 3:** Changes in mucosa-associated microbiota of patients with CD at different time points of follow-up compared to baseline.

**Studies**	**Wright et al**.	**Sokol et al**.	**Neut et al**.		**Machiels et al**.	**Mondot et al**.	**Total** ***↑↓***	
Time point (m) / sample	6/M	6–12/M	3/M	12/M	6/M	6/M	3–12/M	
**α-Diversity**								
Richness	N	N	–	–	–	N	N = 3	
Diversity	N	N	–	–	–	N	N = 3	
**β-DIVERSITY**	D	D	–	–	N	D	D = 3	
**PHYLUM**								
Actinobacteria	↓				↓	↓	0	3
Bacteroidetes	↑	↓				↑	2	1
**FAMILY**							
*Bifidobacteriaceae*	↓				↓		0	2
*Clostridiaceae*	↓					↓	0	2
*Lachnospiraceae*	↑	↑				↑	3	0
*Pseudomonadaceae*	↓					↓	0	2
*Sphingomonadaceae*	↓	↓				↓	0	3
*Staphylococcaceae*					↓	↓	0	2
*Streptococcaceae*	↓	↓				↓	0	3
**GENERA**								
*Anaerostipes*	↑	↑					2	0
*Bacteroides*	↑		↑	↑			2	0
*Bifidobacterium*	↓		↑	↑	↓		1	2
*Blautia*		↑				↑	2	0
*Clostridium*	↑		↑	↑	↑		3	0
*Dialister*		↓			↑		1	1
*Dorea*		↑				↑	2	0
*Enterococcus*		↓	↑	↑			1	1
*Fusobacterium*			↑	↑	↑		2	0
*Haemophilus*	↓	↑					1	1
*Lachnoclostridium*		↑					1	0
*Lactobacillus*	↓						0	1
*Mesorhizobium*		↓				↓	0	2
*Prevotella*			↑	↑			1	0
*Pseudomonas*	↓					↓	0	2
*Roseburia*	↑	↑					2	0
*Ruminococcus*			↑	↑			1	0
*Sphingomonas*		↓				↓	0	2
*Staphylococcus*					↓	↓	0	2
*Streptococcus*	↓	↓				↓	0	3
*Turicibacter*	↓	↓				↓	0	3

*Bacteria alterations at different taxonomic levels (phylum, family, and genera) are displayed in the table*.

*↑ = higher α-diversity or bacteria are more abundant, in CD patients following surgery compared to baseline; ↓ = lower α-diversity or bacteria are less abundant, in CD patients following surgery compared to baseline; D = difference in β-diversity between CD patients following surgery compared to baseline. N = no difference in β-diversity. – = no information provided. Red box represents an increased level, green box represents a decreased level, and empty box represents that no comparison was reported in the included studies*.

*m, month; M, mucosa*.

At the phylum level, CD patients had reduced Actinobacteria and elevated Fusobacteria levels during the postsurgical disease course, while the Bacteroidetes levels were inconclusive in three studies. Following resection, the mucosa of CD patients was enriched with members of the *Lachnospiraceae* family; on the contrary, *Bifidobacteriaceae, Clostridiaceae, Pseudomonadaceae, Sphingomonadaceae, Staphylococcaceae*, and *Streptococcaceae* levels in these patients decreased. No clear overall conclusion could be drawn from the included studies in terms of the bacterial genera populations. Post-operative CD patients had higher relative abundances of the *Anaerostipes, Bacteroides, Blautia, Clostridium, Dorea, Fusobacterium, Prevotella, Roseburia, Ruminococcus*, and *Lachnoclostridium* genera and lower abundances of the *Lactobacillus, Mesorhizobium, Pseudomonas, Sphingomonas, Staphylococcus, Streptococcus*, and *Turicibacter* genera. Moreover, higher Enterococcaceae and *Fusobacterium* and lower Lachnospiraceae and *Faecalibacterium* were found in both post-operative mucosal and fecal microbiota in CD patients. Changes in *Bifidobacterium, Dialister, Enterococcus*, and *Haemophilus* were inconsistent among the included studies. A detailed description of gut microbiota alterations at the phylum, class, order, family, and genus levels in post-operative CD patients is provided in Supplementary Table 4. The fecal microbiota profiles of CD patients were assessed before and after surgery in two longitudinal studies. Strömbeck et al. found that the fecal microbiota composition at an early follow-up (3–10 weeks) after resection is similar to that at a 1-year follow-up (25), while Hamilton et al. demonstrated that fecal bacterial communities are associated with the protection from and the occurrence of CD recurrence after surgery (24).

### Gut Microbiota Profiles of CD Patients at Post-operative Follow-Ups

The gut recolonization and details of the bacterial diversity and composition of CD patients that had undergone surgical intervention were compared with those of healthy control subjects at different time points during follow-up periods in three studies. Wright et al. reported that ileal specimens that were obtained from CD patients 6 and 18 months post-operatively had decreased alpha diversity compared to the control samples (30). In addition, the microbial composition of mucosal and fecal samples differed significantly between CD patients (at the post-operative follow-up) and healthy control subjects, which was reported in two studies.

A comparison of the gut microbial communities, including taxonomic changes and abundances in post-operative CD case and control subjects, is shown in Supplementary Table 5. The fecal microbiota composition in CD patients at the 1-year follow-up only had higher *Ruminococcus gnavus, Shigella* spp., and *Escherichia* spp. levels, while the majority of the bacterial taxa were less abundant than those in healthy subjects. Neut et al. identified and quantified specific bacterial populations in the mucosa-associated microbiota in CD patients that had undergone intestinal resection, using a traditional culture-based method; the authors demonstrated that *Bifidobacterium, Eubacterium*, and *Ruminococcus* populations were rarely encountered in the CD biopsy specimens collected after ileocolectomy at the 3-months and 1-year follow-ups, compared to the ileocolectomy controls (35). The authors of another study with a larger sample size reported that the mucosal biopsy samples that were obtained 6 and 18 months post-operatively from CD patients differed significantly from those of surgical controls; more specifically, they detected increased Fusobacteria, *Bifidobacteriaceae, Enterococcaceae, Bifidobacterium, Fusobacterium*, and *Trabulsiella* counts (30).

### Disease Recurrence-Related Microbiota at the Time of Resection and at Follow-Up

Numerous studies have indicated that distinct gut microbiota profiles at the time of surgery and during the post-operative follow-up are associated with disease recurrence and remission, respectively. As for post-operative endoscopic recurrence-related microbiota, no clear overall conclusion could be drawn from the included studies; however, a number of differences were observed between recurrence cases and those without recurrence when comparing the relative abundance of bacterial taxa (Table 4). Bacteria taxa from mucosal biopsies obtained at the time of resection and associated with PR risk were examined in seven studies, while feces-associated microbiota only examined in one study. The counts of the Bacteroidetes and Firmicutes phyla in CD patients with PR decreased, while those of the Actinobacteria, Fusobacteria, and Proteobacteria phyla increased. The counts of the *Erysipelotrichaceae* and *Lachnospiraceae* families in CD patients with PR were significantly lower, while those of the *Enterobacteriaceae* family increased. Several studies reported that the relative abundance of the *Clostridium, Corynebacterium, Dialister, Enterococcus, Fusobacterium, Lactobacillus, Lactococcus, Proteus, Streptococcus*, and *Veillonella* genera in CD patients with PR increased, while that of the *Alistipes, Bacteroides, Bifidobacterium, Blautia, Coprobacillus, Dorea, Faecalibacterium, Gemella, Odoribacter, Parabacteroides, Paraprevotella, Ruminococcus, Subdoligranulum*, and *Turicibacter* genera decreased. However, the disease recurrence-associated changes in the *Actinomyces* and *Streptococcus* counts were inconsistent among the included studies. Additionally, the microbial communities of fecal samples that were obtained before surgery revealed *Atopobium, Corynebacterium, Gemella*, and *Rothia* counts in CD patients that developed PR than in those that did not. Nine studies provided detailed microbiota profiles associated with recurrence and remission observed at a post-operative colonoscopy, and three studies investigated the fecal microbiota composition after surgery. The findings regarding the mucosa-associated microbiota at the time of surgery revealed that the patients with PR experienced increases in their Fusobacteria and Proteobacteria counts and decreases in their Bacteroidetes and Firmicutes counts. Moreover, CD patients experiencing PR had decreased *Alistipes, Atopobium, Bacteroides, Bifidobacterium, Blautia, Dialister, Dorea, Faecalibacterium, Odoribacter, Parabacteroides, Paraprevotella, Ruminococcus, Subdoligranulum*, and *Turicibacter* counts and increased *Clostridium, Collinsella, Coprobacillus, Enterococcus, Fusobacterium, Proteus*, and *Streptococcus* counts. However, there was no consensus among studies on how the *Odoribacter* and *Oscillospira* counts differed in CD patients with PR. Strömbeck et al. detected higher Actinobacteria and lower Alistipes counts in the fecal microbiota of the PR group at their 1-year follow-up; Alistipes was found to correlate negatively with the Rutgeerts score (25). In another study tracking the trends in the fecal microbiota changes in patients with endoscopic recurrence, the relative Fusobacterium and Bifidobacterium abundances increased and decreased, respectively, in 1, 3, and 6 months after surgery compared to that in patients in remission (26). In addition, Hamilton et al. found that bacterial clusters enriched with *Enterobacteriaceae* and *Lachnospiraceae* were associated with an increased risk of disease recurrence and the maintenance of remission, respectively (24).

**Table 4:** Recurrence-associated fecal or mucosal microbiota at the time of resection and post-operative follow up.

**Studies**	**Hamilton et al**.		**Strömbeck et al**.	**Wright et al**.		**Sokol et al**.		**Dey et al**.	**Machiels et al**.					**Cruz et al**.		**Mondot et al**.	**Sokol et al**.		**Keshteli et al**.	**Laffin et al**.	

**Time point Sample**	**6 m (F)**	**18 m (F)**	**12 m (F)**	**6 m (M)**	**18 m (M)**	**0 m (M)**	**6–12 m (M)**	**0 m (M)**	**0 m (M)**	**0 m (F)**	**1 m (F)**	**6 m (M)**	**6 m (F)**	**0 m (M)**	**6 m (M)**	**6 m (M)**	**0 m (M)**	**6 m (M)**	**0 m (M)**	**0 m (M)**	**6 m (M)**
**PHYLUM**																					
Actinobacteria			**↑**																	**↑**	
Bacteroidetes																	**↓**	**↓**	**↓**	**↓**	**↑**
Firmicutes									**↓**								**↓**	**↓**		**↓**	**↓**
Fusobacteria												**↑**	**↑**						**↑**		
Proteobacteria					**↑**		**↑**												**↑**	**↑**	
**FAMILY**																					
*Actinomycetaceae*							↓		↓												
*Bacteroidaceae*																↓			↓	↓	↑
*Enterobacteriaceae*																			↑	↑	
*Enterococcaceae*		↑					↑									↑					
*Erysipelotrichaceae*								↓	↓												
*Lachnospiraceae*		↓					↓	↓												↓	↓
*Peptostreptococcaceae*	↓									↓		↑									
**GENERA**																					
*Actinomyces*									↓					↑							
*Alistipes*			↓											↓	↓						
*Atopobium*					↓					↑											
*Bacteroides*														↓	↓	↓					
*Bifidobacterium*			↓										↓		↓		↓	↓			
*Blautia*							↓							↓							
*Clostridium*														↑	↑						
*Collinsella*							↑							↓							
*Coprobacillus*										↓		↑		↓							
*Corynebacterium*						↑				↑				↑							
*Dialister*							↓							↑	↓	↓					
*Dorea*							↓							↓		↓					
*Enterococcus*							↑							↑	↑	↑					
*Faecalibacterium*				↓	↓									↓			↓	↓			
*Fusobacterium*												↑	↑	↑							
*Gemella*										↑				↓							
*Lachnobacterium*					↓																
*Lactobacillus*														↑							
*Lactococcus*														↑							
*Odoribacter*					↓							↑		↓	↓						
*Oscillospira*					↓							↑									
*Parabacteroides*														↓	↓						
*Paraprevotella*					↓							↑		↓							
*Proteus*				↑	↑									↑							
*Ruminococcus*						↓	↓		↓					↓	↓	↓					
*Streptococcus*						↑			↓					↑	↑						
*Subdoligranulum*														↓	↓						
*Turicibacter*					↓									↓							

*Only bacteria, for which at least two studies have reported the alterations at different taxonomic levels (phylum, family, and genera), are displayed in the table*.

*↑ = bacteria are more abundant in CD patients with post-operative endoscopic recurrence compared to those in remission; ↓ = bacteria are less abundant in CD patients with post-operative recurrence compared to those in remission. Red box represents an increased level, green box represents a decreased level, and empty box represents that no comparison was reported in the included studies*.

*m, month; M, mucosa; F, feces*.

### Predictive Potential of Microbial Factors in the PR of CD

The predictive potential of microbial factors at the time of surgery and at the time of post-operative endoscopic evaluation was further evaluated to guide disease diagnosis and treatment (Table 5). However, no consistent differences were detected in the counts of specific bacteria, which would allow their use in predicting endoscopic recurrence. Wright et al. reported that microbial analysis of the ileal mucosa, which takes into account the presence of Proteus, abundance of Faecalibacterium, and smoking status, at 6 and/or 18 months post-operatively, is moderately accurate in predicting endoscopic recurrence (30). In addition, increased *Corynebacterium* and decreased *Ruminiclostridium 6* counts at baseline were identified as predictive factors of endoscopic recurrence for CD patients (27). Machiels et al. reported that the *Ralstonia, Haemophilus, Gemella*, and *Phascolarctobacterium* abundances in resected specimens were good predictors using C5.0 classification tree analyses or random forest models (26). However, the considerable predictive power of *Coprobacillus, unidentified Lachnospiraceae*, and *Dorea* obtained from fecal samples before surgery could not be confirmed after validation using the forest model. Furthermore, Keshteli et al. found that the concentrations of urinary 1,6-anhydro-beta-D-glucose, L-3,4-dihydroxyphenylalanine, propylene glycol, and ethylmalonate were related to CD recurrence after ileocolonic resection, with an associated area under the curve value of 0.91 (95% CI: 0.73–1.00) (28).

**Table 5:** Specific gut microbiota and metabolites as predictor of post-operative endoscopic recurrence at the time of resection and post-operative follow-up.

**Studies**	**Time point (sample)**	**Model**	**AUC**
Wright et al.	6/18 m (M)	*Proteus, Faecalibacterium*, smoking status	0.74 (95% CI 0.69–0.79)
Sokol et al.	0 m (M)	*Corynebacterium, Ruminiclostridium 6*	RF: 0.81 (95% CI 0.61–1.00)
Machiels et al.	0 m (M)	*Ralstonia, Haemophilus, Gemella, Phascolarctobacterium*	C5.0: 0.738; RF: 1.00
	0 m (F)	*Coprobacillus, unidentified Lachnospiraceae, Dorea*	C5.0: 0.79; RF: 0.50
Keshteli et al.	0 m (U)	Levoglucosan, L-DOPA, propylene glycol, ethylmalonate	MCCV: 0.71 (95% CI 0.73–1.00)

*m, month; M, mucosa; F, feces; U, urine; AUC, area under the curve; CI, confidence interval; RF, random forest model; MCCV, Monte-Carlo cross validation; levoglucosan, 1,6-anhydro-beta-D-glucose; L-DOPA, L-3,4-dihydroxyphenylalanine*.

### Microbiota-Based Therapies to Prevent the PR of CD

The recolonization of the intestinal tract by gut microbiota plays a critical role in determining whether there will be post-operative relapse at the resection site. This indicates that interventions that aim at manipulating the microbiome should, in theory, have an integral role in the prevention of PR for CD patients. However, there is a lack of evidence-based recommendations on this topic.

As is shown in Table 6, five studies (three comparing metronidazole with a placebo, one comparing ornidazole with a placebo, one comparing ciprofloxacin with a placebo) evaluated the efficacy of antibiotics in preventing post-operative endoscopic or clinical recurrence in CD patients. Based on the available data, nitroimidazole antibiotics (metronidazole, ornidazole) are effective in preventing the clinical and endoscopic PR of CD compared to placebo drugs, which may be the most cost-effective option. In the first study, Rutgeerts et al. demonstrated that 3-month-long metronidazole therapy significantly decreased the severe endoscopic recurrence incidences (13 vs. 43%, *P* = 0.02) in the neoterminal ileum. Additionally, this treatment seemed to delay symptomatic recurrence; however, it was associated with a high incidence of side effects (40). Nevertheless, there were no significant differences between the metronidazole treatment and the placebo treatment in terms of reducing the clinical PR at the 1-year follow-up. In a subsequent study, the same authors found that taking ornidazole at a dose of 1 g/day for a year is effective in preventing PR at the 3-months and 1-year follow-ups, while no significant differences in terms of clinical recurrence were observed at subsequent follow-ups (39). The results of another recent study showed that taking low doses of metronidazole (250 mg three times per day) for 3 months decreases significantly endoscopic PR rates in CD patients within 12 months and is well-tolerated (35). However, Mañosa et al. showed that the risk of endoscopic recurrence is not reduced significantly with the combined use of metronidazole and azathioprine compared to the sole use of azathioprine; however, the use of metronidazole does not worsen azathioprine's safety profile (37). Yet in spite of this, the results of another randomized controlled trial demonstrated that the long-term addition of azathioprine to a post-operative 3-months course of metronidazole is more effective than using metronidazole alone (49). Moreover, a 6-months course of ciprofloxacin is not more effective than using a placebo drug in terms of preventing PR in CD patients who underwent surgery, and a high proportion of patients discontinue their treatment because they cannot tolerate it well (38).

**Table 6:** Summary of studies of antibiotic to prevent post-operative recurrence in patients with CD.

**Study**	**Year**	**Group, Number**	**ABX, Doses**	**Treatment duration**	**Recurrence definition**	**Recurrence score**	**Follow-up**	**ER rate (%)**, ***P*****-value**				**CR rate (%)**, ***P*****-value**			
Glick et al.	2019	TX, *N =* 35 PBO, *N =* 35	Metronidazole 250 mg/tid	3m	ER	Rutgeerts	12 m	12 m: 20 (7/35)	0.01						
								12 m: 54.3 (19/35)							
Mañosa et al.	2013	TX, *N =* 25 PBO, *N =* 25	Metronidazole 15–20 mg/kg per day	3m	ER, CR	Rutgeerts, HBI	6, 12 m	6 m (PP): 22 (5/23)	0.23	6 m (ITT): 28 (7/25)	0.19	6 m (PP): 0 (0/23)	NS		
								6 m (PP): 36 (8/22)		6 m (ITT): 44 (11/25)		6 m (PP): 0 (0/22)			
								12 m (PP): 30 (7/23)	0.15	12 m (ITT):36 (9/25)	0.15	12 m (PP): 4 (1/23)	0.48		
								12 m (PP): 50 (11/22)		12 m (ITT): 56 (14/25)		12 m (PP): 9 (2/22)			
Herfarth et al.	2013	TX, *N =* 17 PBO, *N =* 16	Ciprofloxacin 500 mg/bid	6m	ER, CR	Rutgeerts, HBI	1, 3, 6m	6 m (PP): 42 (3/7)	0.61	6 m (ITT): 65 (11/17)	0.81	6 m (PP): 22 (2/9)	0.92	6 m (ITT): 12 (2/17)	0.67
								6m (PP): 55 (5/9)		6m (ITT): 69 (11/16)		6m (PP): 20 (2/10)		6m (ITT): 13 (2/16)	
Rutgeerts et al.	2005	TX, *N =* 38 PBO, *N =* 40	Ornidazole 1 g/d	12 m	ER, CR	Rutgeerts, CDAI	3, 12, 24, 36m	3 m: 34.4 (11/32)	0.047			12 m: 7.9 (3/38)	0.005		
								3 m: 58.8 (20/34)				12 m: 37.5 (15/40)			
								12 m: 53.6 (15/28)	0.037			24 m: 29.7 (11/37)	0.17		
								12m: 78.8 (26/33)				24 m: 45 (18/40)			
												36 m: 45.9 (17/37)	0.53		
												36 m: 47.5 (19/40)			
Rutgeerts et al.	1995	TX, *N =* 30 PBO, *N =* 30	Metronidazole 20 mg/kg per day	3 m	ER, CR	Rutgeerts, Symptoms	3, 12, 24, 36m	3 m: 52 (12/23)	0.09			12 m (PP): 4 (1/23)	0.044	12 m (ITT): 7 (2/29)	0.06
								3 m: 75 (21/28)				12 m (PP): 25 (7/28)		12 m (ITT): 25 (7/28)	
												24 m (PP): 28 (6/23)	0.171	24 m (ITT): 24 (7/29)	0.112
												24 m (PP): 43 (12/28)		24 m (ITT): 43 (12/28)	
												36 m (PP): 30 (7/23)	0.13	36 m (ITT): 31 (9/29)	0.117
												36 m (PP): 50 (14/28)		36 m (ITT): 50 (14/28)	
Total		TX, *N =* 145		3–12 m			1–36 m								
		PBO, *N =* 146													

*CD, Crohn's disease; TX, treatment; ASX, antibiotic treatment; PBO, placebo; ER, endoscopic recurrence; CR, clinical recurrence; HBI, Harvey–Bradshaw Index; CDAI, Crohn's Disease Activity Index; ITT, intention-to-treat; PP, per-protocol; m, month; NS, no significance*.

Another method of gut microbiota manipulation is by using a supplement of live and safe microbes that restore the beneficial intestinal microbial flora; in this case, the use of a probiotic formulation may be an appealing alternative. Seven studies examined the effect of probiotics on preventing the PR of CD; however, none of these studies detected a significant effect of probiotics on clinical or endoscopic recurrence (as is shown in Table 7). Among the included studies, three investigated the ability of VSL#3 (a mixture of eight different bacterial strains), two evaluated the efficacy of *Lactobacillus johnsonii* LA1, one examined the effect of *Lactobacillus rhamnosus* strain GG, and one observed the potency of Synbiotic 2000 (a cocktail of four probiotics and four prebiotics). Nevertheless, the combination of rifaximin and VSL#3 was efficient in preventing the severe endoscopic recurrence of CD after surgical resection, as reported by Campieri et al. (47).

**Table 7:** Summary of studies of probiotics to prevent post-operative recurrence in patients with CD.

**Study**	**Year**	**Group, Number**	**Probiotics, Doses**	**Treatment duration**	**Recurrence definition**	**Recurrence score**	**Follow-up**	**ER rate (%)**	***P*-value**	**CR rate (%)**	***P*-value**
Madsen et al.	2020	TX, *N =* 58 PBO, *N =* 62	VSL#3 1.8 × 10^12^ CFU/d	3 m	ER	Rutgeerts	3 m	9.3 (4/43)	0.36		
								15.7 (8/51)		
Fedorak et al.	2015	TX, *N =* 58 PBO, *N =* 62	VSL#3 1.8 × 10^12^ CFU/d	3 m	ER	Rutgeerts	3 m	39.5 (17/43)	0.3		
								47.1 (24/51)		
Chermesh et al.	2007	TX, *N =* 20 PBO, *N =* 10	Synbiotic 2000 NS	-	ER	Rutgeerts	3, 24 m	-	NS	-	NS
								-		-	
Gossum et al.	2007	TX, *N =* 34 PBO, *N =* 36	LA1 2 g/d (10^10^ CFU/d)	3 m	ER, CR	Rutgeerts, CDAI	3 m	ITT: 46.4 (13/28)	0.158	15 (4/27)	0.91
								ITT: 29.6 (8/27)		13.5 (3/22)	
								PP: 19 (5/27)	0.054		
								PP: 9 (2/22)			
Marteau et al.	2006	TX, *N =* 48 PBO, *N =* 50	LA1 4 × 10^9^ CFU/d	6 m	ER, CR	Rutgeerts, CDAI	6 m	ITT: 49 (21/43)	0.15	9.3 (4/43)	0.45
								ITT: 64 (30/47)		6.3 (3/47)	
								PP: 49 (17/35)	0.21		
								PP: 63 (27/43)			
Prantera et al.	2002	TX, *N =* 23 PBO, *N =* 22	Lactobacillus GG 1.2 × 10^10^ CFU/d	12 m	ER, CR	Rutgeerts, CDAI	12 m	60 (9/15)	0.297	16.6 (3/18)	0.45
								35.3 (6/17)		10 (2/20)	
Campieri et al.	2000	TX, *N =* 20 PBO, *N =* 20	VSL#3 6 g/d (3 × 10^11^ CFU/d)	9 m	ER	Rutgeerts	12 m	20 (4/20)	0.15		
								40 (8/20)			
Total		TX, *N =* 261		3–12 m			3–24 m				
		PBO, *N =* 262									

*CD, Crohn's disease; TX, treatment; PBO, placebo; ER, endoscopic recurrence; CR, clinical recurrence; CDAI, Crohn's Disease Activity Index; ITT, intention-to-treat; PP, per-protocol; m, month; NS, no significance*.

## Discussion

There is limited consensus on the gut microbiota profiles of CD patients at the time of surgical resection and at the post-operative follow-up. Understanding the microbial communities associated with PR has the potential of improving the therapeutic options for CD patients that have undergone intestinal resection.

In this systematic review, we initially evaluated whether CD patients had a distinct microbiota composition at the time of surgery compared to healthy controls. As has been shown in previous studies, the majority of the studies included in this review suggested that the ileal mucosa-associated microbiota in CD patients exhibited reduced bacterial richness and diversity, while there was a clustering of samples with statistically significant differences between CD patients and healthy controls. Only one study from Machiels et al. simultaneously analyzed preoperative mucosal and fecal microbiota and found that CD patients had distinct characteristics of mucosal and fecal microbiota before surgery, which further confirmed that fecal and mucosal microbiota constitute different ecological environments (26). Although not even a single bacterial taxon had consistently altered counts across all included studies, we identified bacterial taxa obtained from surgical specimens that allowed us to discriminate between CD patients and healthy subjects, in several studies. Among the bacterial taxa reported to have altered relative abundances in CD cases, the Bacteroidetes and Firmicutes phyla were significantly less represented, while the Proteobacteria phylum was significantly more represented in mucosal microbiota, which corroborates the fecal microbiota findings. In addition, the expansion of Fusobacteria, a putative aggressive phylum, was observed in the surgical biopsies. A number of factors could influence the bacterial populations following ileocolonic resection, including substantial catabolic stress, retrograde flow of colonic contents, inflammatory changes involved in intestinal wound healing, and altered immune function (50, 51). At the time of post-operative endoscopy, the population of Actinobacteria, a proteolytic bacterial phylum with the capacity to invade and exacerbate inflammation, was depleted in patients with CD; however, the phylum Fusobacteria maintained its higher relative abundance, while the alterations in the phylum Bacteroidetes count were inconsistent. Even so, higher abundance of Enterococcaceae and *Fusobacterium* and lower abundance of Lachnospiraceae and *Faecalibacterium* existed in both post-operative mucosal and fecal microbiota in CD patients.

The counts of many known gut pathogens (*Enterococcus, Escherichia, Fusobacterium, Streptococcus, Trabulsiella*, and *Veillonella*) increased in the inflamed resection specimens, whereas the counts of essential types of butyrate and other short-chain fatty acid (SCFA)-producing bacteria were observed, such as *Faecalibacterium, Blautia, Clostridium, Coprococcus, Lachnobacterium, Lachnospira*, and *Ruminococcus* spp. In addition, baseline samples with enriched facultative anaerobic and oxygen-tolerant bacteria likely reflect active inflammation. The mechanisms through which these mucosa-associated bacteria that are involved in CD affect the intestinal permeability are by increasing the ability of the bacteria to adhere to the intestinal epithelium, inducing inflammatory responses by regulating the expression of inflammatory genes, restricting epithelial cell growth and differentiation by restricting energy sources, promoting invasion by pathogenic bacteria by destroying the intestinal mucus, weakening the anti-inflammatory functions by altering regulatory T cell differentiation, and influencing IBD-related genetic risk variants by altering the abundance of gut microbiota (52–58).

Numerous studies have documented that alterations in gut microbial profiles at the time of surgery or at the post-operative follow-up are linked to the post-operative disease course in CD patients. As fecal and mucosal-associated microbiota constitute different ecological environments, they have different alteration trends (59). Notably, the presence of mucosal bacterial genera, such as *Bacteroides, Prevotella*, and *Parabacteroides*, which are associated with saccharolytic metabolism, has been correlated with increased remission compared to the presence of bacterial genera, such as *Enterococcus* and *Veillonella*, which are associated with fermentation and lactic acid production. However, no specific bacterial taxa are consistently different between CD patients with or without PR in any of the included studies. Among the bacteria reported to have increased relative abundance in CD patients with PR, several genera have previously been associated with triggering host inflammatory responses (26, 60, 61). Conversely, several commensal bacteria with lower relative abundance are known to exert an anti-inflammatory effect by decreasing proinflammatory colonic pro-inflammatory cytokine synthesis and inducing anti-inflammatory cytokine secretion (33). In addition, patients with recurrent CD retain microbiota that favors proteolytic-fueled fermentation and lactic acid production, while CD patients in remission retain a predominantly saccharolytic and SCFA-producing microbiota (32). Moreover, altered bacteria involved in hydrogen sulfide production or a specific enzymatic machinery associated with the metabolism of bile acids are involved in the post-operative disease course of CD patients (31, 62, 63). Furthermore, changes in the ecology of depleted SCFA-producing bacteria may permit the expansion of pathogenic bacteria through luminal environmental perturbation (24). To date, no study has evaluated the role of the metabolomic profiling of gut microbiota at the time of surgery or at the post-operative follow-up in the identification of metabolites that may be associated with CD recurrence after intestinal resection.

As there were differences between CD patients with PR and those without in terms of their gut microbiota alterations, there may be a window of opportunity for microbial biomarkers to predict and monitor the post-operative disease outcome. Moreover, equipping clinicians with the prognostic biomarkers that will allow them to identify patients more likely to experience PR will reduce the duration of the drug treatment that is unlikely to be beneficial. However, the number of published studies investigating the involvement of gut microbiota both in monitoring the post-operative disease progression and in assessing the response of CD patients to a treatment is surprisingly low. In this review, we identified only four studies that provide information on the potential of gut microbiota to predict the PR of CD, but their results were too heterogeneous to allow us to reach confident conclusions regarding a microbial biomarker. Among the most discriminative features, the high abundance of bacteria from the Proteobacteria phylum (e.g., *Proteus* and *Ralstonia*) as well as in *Ruminiclostridium gnavus* (Gammaproteobacteria) and *Corynebacterium*, and the reduced abundance of several members of the Firmicutes phylum, particularly the *Lachnospiraceae* and the *Ruminococcaceae* families (e.g., *Faecalibacterium, Gemella, Phascolarctobacterium, Coprobacillus, unidentified Lachnospiraceae*, and *Dorea*), were predictive of endoscopic PR. Furthermore, adding the usual clinical risk factors (e.g., smoking status) in the prediction model may improve its diagnostic efficiency and accuracy regarding the gut microbiota of interest. As reported by Keshteli et al., distinctive urinary metabolomic profiling associated with Bacteroidales and Gammaproteobacteria has the potential to be used as a biomarker for the identification of CD patients who develop endoscopic disease recurrence after ileocolonic resection (28). The currently available studies are insufficient in elucidating the prevalence, diversity, distribution, and function of the identified bacteria associated with PR. Further research is required to confirm and define the sensitivity and specificity for recurrence or remission of such bacterial profiles using larger patient cohorts and more targeted bacterial analysis. Moreover, functional analyses are required to characterize the phylogenetic alterations at the gene expression level.

Various pharmaceutical treatments have been developed in an attempt to prevent or delay potential recurrence and subsequent surgery. However, the strategy directly targeted to gut microbiota disturbance is currently quite limited. While studies have attempted to utilize antibiotics or probiotics in an effort to prevent the PR of CD, their results are non-conclusive. Antibiotics can potentially ameliorate the microbial environment of patients suffering from IBD, both by decreasing the counts of pro-inflammatory bacteria and by increasing those of beneficial ones, throughout the intestinal lumen (64). Funayama et al. reported that antibacterial treatment was useful in post-operative CD patients whose assessments were complicated by bacterial overgrowth (65). Nitroimidazole antibiotics have been proven to be effective in preventing the clinical (RR: 0.23; 95% CI: 0.09–0.57; NNT: 4) and endoscopic (RR: 0.44; 95% CI: 0.26–0.74; NNT: 4) PR of CD, but the high rate of patients that are intolerant to them and their questionable long-term effects beyond the end of the treatment preclude their widespread use. Recent promising data on the activity of the non-absorbable antibiotic rifaximin (which is generally well-tolerated) in CD patients suggest that assessment of this agent in reducing post-operative recurrence is warranted. Probiotic supplements have been used successfully in the prevention of pouchitis and the maintenance of remission in active ulcerative colitis; nevertheless, the present review demonstrates that studies to date have failed to identify any benefit of probiotic supplement administration in preventing the PR of CD (66–69). Intriguingly, Campieri et al. reported that the combination of a non-absorbable antibiotic (rifaximin) and a highly bacterial concentrated probiotic (VSL#3) is efficient in preventing the severe endoscopic recurrence of CD (47). However, none of the studies examined in the present review investigated the therapeutic mechanisms of either antibiotics or probiotics on gut microbiota modification. Several mechanisms mediate the therapeutic action of antibiotics and probiotics, such as the inhibition of pathobionts, increase in beneficial bacteria, modification of bacterial metabolites, regulation of immunity, improvement in the mucosal barrier, or absorption of toxic substances (70–75). The outcomes of microbial-based treatments indicate that the possibility of using combination-based strategies, such as the early post-operative use of antibiotics to prevent pathogenic recolonization followed by maintenance with the use of probiotics to establish a durable anti-inflammatory post-operative microflora, may yield the greatest benefit with the least risk of disease recurrence in CD patients. Unfortunately, the efficacy of fecal microbiota transplants in preventing the PR of CD remains unknown.

There are some limitations to this review that warrant discussion. It is well-known that several factors may exert an influence on the composition of gut microbiota, including geographic, cultural, demographic, dietary, and preoperative and post-operative medications differences, which may explain some of the observed discrepancies among the different studies (76, 77). In addition, other risk factors associated with disease recurrence failed to be addressed in this review, which perhaps explain some of the discrepancies between the results. Furthermore, the included studies consisted of 10 retrospective studies and two prospective studies, which might be one possible reason for the inconsistencies. Lastly, differences in the methodology used, such as specimen type, sample storage methods, DNA extraction methods, primers targeting different regions, bioinformatic pipelines, and reference databases used, may explain the heterogeneous results observed in the examined studies.

## Conclusion

In this systematic review, we characterized the mucosa-associated microbiota at the time of surgery and the profiles of the bacteria that recolonized the intestinal tract following resection. Additionally, we highlighted specific bacterial taxa, the counts of which either increased or decreased and that are associated with the endoscopic PR of CD. Although consistent recurrence-associated gut microbiota with predictive value could not be identified from the examined studies, a few microbial predictors were suggested. CD patients with PR tend to gain pathogenic bacteria with a pro-inflammatory effect and lose SCFA-producing bacteria. The gut microbiota manipulation through the administration of either antibiotics or probiotics may not offer a promising alternative in the prevention of PR in patients with CD. Future research should focus on investigating differences in the function and composition of the gut microbiota associated with PR and post-operative remission. Additionally, the use of larger patient cohorts is recommended to confirm the sensitivity and specificity of bacterial profiles with predictive value. Furthermore, effective microbial-based therapies based on an individual patient's microbial profile that are used to prevent PR and can be administered for prolonged time periods with acceptable side effects are urgently awaited.

## Data Availability Statement

The original contributions presented in the study are included in the article/Supplementary Materials, further inquiries can be directed to the corresponding author/s.

## Author Contributions

MC guarantor of the article. MC and XZ designed the study. XZ wrote the manuscript. ZT and XL collected the data. MZ, NL, and SX analyzed the data. RM, ZZ, and RF revised the manuscript. All authors approved the final version.

## Conflict of Interest

The authors declare that the research was conducted in the absence of any commercial or financial relationships that could be construed as a potential conflict of interest.

## Abbreviations

CD: Crohn's disease
IBD: Inflammatory bowel disease
PR: Post-operative recurrence
SCFA: Short chain fatty acid.
